# Optimizations for the EcoPod field identification tool

**DOI:** 10.1186/1471-2105-9-150

**Published:** 2008-03-17

**Authors:** Aswath Manoharan, Jeannie Stamberger, YuanYuan Yu, Andreas Paepcke

**Affiliations:** 1 Department of Computer Science, Stanford University, Stanford, CA 95305, USA; 2 Department of Biology, Stanford University, Stanford, CA 95305, USA

## Abstract

**Background:**

We sketch our species identification tool for palm sized computers that helps knowledgeable observers with census activities. An algorithm turns an identification matrix into a minimal length series of questions that guide the operator towards identification. Historic observation data from the census geographic area helps minimize question volume. We explore how much historic data is required to boost performance, and whether the use of history negatively impacts identification of rare species. We also explore how characteristics of the matrix interact with the algorithm, and how best to predict the probability of observing a previously unseen species.

**Results:**

Point counts of birds taken at Stanford University's Jasper Ridge Biological Preserve between 2000 and 2005 were used to examine the algorithm. A computer identified species by correctly answering, and counting the algorithm's questions. We also explored how the character density of the key matrix and the theoretical minimum number of questions for each bird in the matrix influenced the algorithm. Our investigation of the required probability smoothing determined whether Laplace smoothing of observation probabilities was sufficient, or whether the more complex Good-Turing technique is required.

**Conclusion:**

Historic data improved identification speed, but only impacted the top 25% most frequently observed birds. For rare birds the history based algorithms did not impose a noticeable penalty in the number of questions required for identification. For our dataset neither age of the historic data, nor the number of observation years impacted the algorithm. Density of characters for different taxa in the identification matrix did not impact the algorithms. Intrinsic differences in identifying different birds did affect the algorithm, but the differences affected the baseline method of not using historic data to exactly the same degree. We found that Laplace smoothing performed better for rare species than Simple Good-Turing, and that, contrary to expectation, the technique did not then adversely affect identification performance for frequently observed birds.

## Background

Bio-diversity researchers study how the abundance and geographic distribution of organisms change in response to varying environmental conditions. These studies rely heavily on observations of species acquired in the field and spanning many different geographical regions. Observations include data such as species presence/absence, counts of individuals, gender, life history stages (egg, juvenile, adult), behaviour (e.g., feeding), time and location where observations were made, photographs, audio recordings and environmental conditions where the observations occurred.

Species richness (number of species) and abundance (population size) data are crucial for understanding processes that govern the distribution of species across space. Quantifying beta-diversity (species turn-over in space) and endemism (species that exist only in one place) is critical for conservation biology [1]. This discipline includes developing conservation schemes [2], such as identifying biological 'hotspots', which are areas with high levels of species richness. Monitoring abundance is critical for assessing species health, because population losses threaten species diversity [3], and for understanding sensitivity of population size to environmental factors [4], including climate change [5]. All these issues are currently hotly debated in the ecology literature. See for example Ostling 2005 describing controversy around the neutral theory, Hubbell 2001 vs. McGill Hadly and Maurer 2005, and Graves and Rahbek 2005. More temporal and spatially explicit data on species and population sizes will inform these debates.

Traditionally, bio-diversity researchers have relied on their own collections of observation data to carry out their analyses, and such collections are at inherently limited temporal and spatial resolution. Better informed analysis can be performed on larger quantities of data gathered across many different geographical regions.

One way to achieve larger datasets for scientific analyses is to enlist the help of the public, amateurs and volunteers, in gathering species observation data. The possible numbers of participants covering a wide geographic area in a short period of time is exemplified by the Christmas Bird Count (CBC), which in its 105^th ^event of 2004 – 2005 had 56,623 participants conduct 2,200 counts, recording almost 70 million birds over a period of a few weeks in Canada, the United States, Latin America and the Caribbean [6]. Volunteers defray otherwise prohibitive costs of surveys [7] and generously donate travel expenses [8].

Researchers, however, are wary of using amateur observations as the basis of their studies, in part because of concerns over accuracy of species identifications [9]. One way to increase accuracy is to provide tools that are intuitive to use, which limit the number of mistakes an amateur can make, allow a user to enter evidence for a reviewer to later verify the observation, and provide an overall pleasant user experience [8].

### Potential improvements to the process

Traditional paper-based field guides and dichotomous taxonomic trees typically arrange species according to morphological commonalities, reflecting their phylogenetic relationships, rather than their geographic distribution or abundance. In practical use, species found in the same location may be morphologically different and only distantly related. Consequently, morphologically organized guides require readers to access many parts of the book. Also, the number of species found in any one area is small relative to the number of species in a typical regional field guide. The number of species and size of an area are in fact related in a log/log fashion [10].

Local field guides are taxonomic keys limited to only the species found in one area. Unfortunately, local guides are expensive and time consuming to create. And they may not contain newly arrived or rare species. The impact of abundance on species identification was examined in [11].

Building on the existing idea of interactive keys, we constructed a tool, EcoPod [12], that attempts to combine the completeness of general field guides with advantages of their local counterparts. Ideally such a tool would reduce the number of questions the user needs to answer for identifying common or previously observed species, while not penalizing identification of rare species by increasing the number of questions required to ID them.

### EcoPod

EcoPod has an intuitive user interface and includes features such as an audit trail that allows users to document all decisions with supporting materials, like audio recordings, notes, or photographs. Users can also indicate whether they are certain about their decision on the state of each character. Experts can use this auxiliary information later while reviewing identifications. Such quality checks can then improve confidence in an evolving dataset of observations.

**Figure 1:**
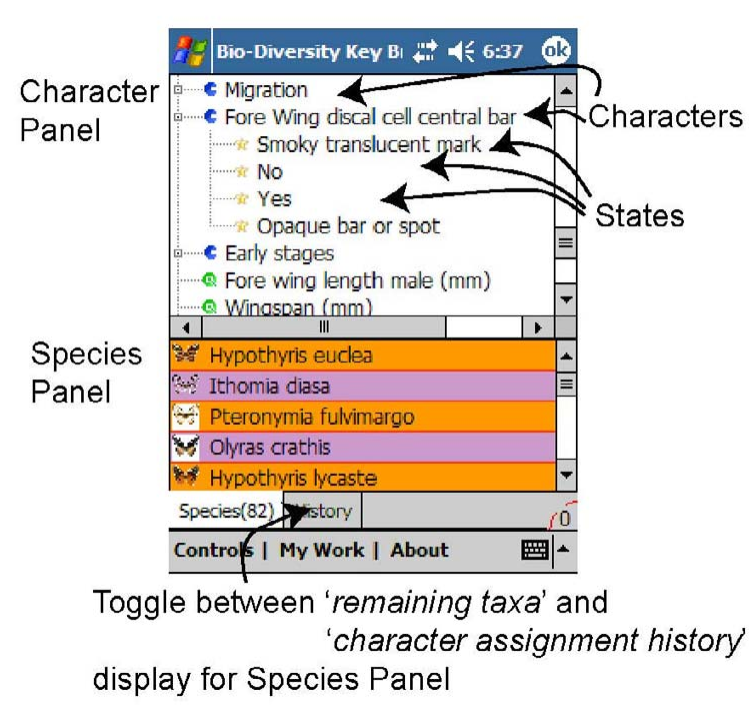
Main EcoPod screen; a device for organism identification.

EcoPod presents the operator with a list of characters. As the user specifies the respective states, the remaining taxa are narrowed down and listed. The most discriminating characters are displayed at the top of the list at all times (see Figure 1), similar to algorithms introduced by [13] and also found in desktop species identification programs *Delta *[11,14,15] and [16].

We have previously described EcoPod's user interface in detail [12]. An exploration of the character ordering is the focus of the work we present here. The current system (internally) conceptually begins with a matrix view of the taxonomy. From this matrix the system uses information gain theory to derive a *decision tree *that controls the user interface and is equivalent to a dichotomous key.

For illustration, Table 1 shows a highly simplified matrix of four birds, a Murres, Egret, Gray Jay, and Turkey.

**Table 1:** Partial matrix view of four bird characters

	**Primary Color**	**Slender**	**Long Bill**
**Murres**	White	No	No
**Gray Jay**	Gray	Yes	No
**Egret**	White	Yes	Yes
**Turkey**	Gray	No	No

**Figure 2:**
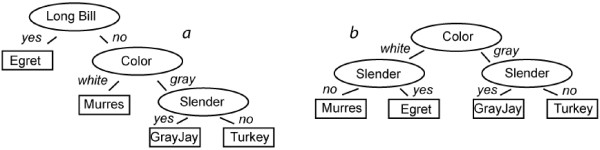
Decision trees for four hypothetical species with theirstates and characters. a) displays the optimal tree for Case 1, where Egrets are most abundant, while b) displays the optimal tree for Case 2, where all four species are equally abundant. Refer to [19] for further details.

From this matrix we can construct multiple decision trees that could guide the computing device's interface. Figure 2 shows two possible trees.

Using the left tree, the device would first ask the user whether the specimen being observed had a long bill. If the bird in question indeed exhibited a long bill, the identification would be accomplished with a single question. The specimen would be an Egret.

However, if we assume that all species of birds are equally likely to occur in the observer's locale, then on average more questions would be required. For example, both Gray Jay and Turkey would need answers to three questions before an identification could be produced. The average number of questions in the left tree is (1+2+3+3)/4 = 2.25. The right hand tree in contrast, produces an average of 2 questions for identifications. Under the assumption of uniform species distribution, the right side tree is therefore optimal. Osborne [17] additionally showed that if the operator of a key has some non-zero probability of answering questions incorrectly, then short keys tend to minimize erroneous identifications.

Currently, EcoPod always uses that uniform distribution decision tree on the right of Figure 2. While for this example EcoPod would never do as well as requiring just a single question to produce an identification, it would perform well on average across the species. The work we present in the remainder of this writing removes the assumption that all species of a census are equally likely to be observed. If, for the above example, the Egret population were overwhelmingly larger than that of the other three species in the geographic area where the tool is to support identifications, EcoPod would be well advised to choose the left-side decision tree of Figure 2. How would the device 'know' about such supporting information? Our vision is for EcoPod to be carried into a field station that makes statistics of prior local species observations available through a wireless network. The portable identification device would absorb these statistics and calibrate its operation such that it would ask users as few questions as possible while they are identifying organisms in the surrounding area. Short of such a vision, historic observation data might be downloaded from the Web prior to commencing field work.

To this end we developed an algorithm that produces decision trees from a taxonomic matrix based on frequency of prior observations. To test this algorithm we acquired observations of 104 bird species from Stanford University's 1200 acre Jasper Ridge Biological Preserve (JBRP). The observations were point counts across the preserve. The counts were repeated multiple times per year between 2000 and 2005. The data included a total of 8200 observations with 10,600 birds counted.

Intuitively, the use of historical information should speed up identification of previously observed organisms. But a number of factors need to be explored to ensure success. First, observations are costly. Beyond their original collection, the data must be curated over many years. Associated computers must be maintained. A realization of the idea thus does not come free of charge, and we must be sure of its value. Second, observations of rare species are valuable and should not be adversely impacted by the new technology.

### Summary of Contributions

We answer the following questions:

• *Efficacy of species abundance data*: How much faster are identifications of common species vs. rare species when using historical abundance data? Does the inherent bias towards common species increase the number of questions to identify rare species over a paper-based dichotomous taxonomic key?

• *Required amount of historical data*: How many years (1–6) of observations are necessary to gain a significant benefit? Does performance depend on a particular year of the observation?

• *Unobserved species*: Computationally, what probability weight should be assigned to a species that has never been observed at the location before (i.e., abundance = 0)? Known smoothing algorithms redistribute probability mass, but their efficacy depends on the corpus of data. Does the choice of two smoothing algorithms, Laplace and Good-Turing, influence the number of questions to detect previously observed as well as 50 never before observed species?

• *Taxonomic key properties*: How strongly does the matrix density, or the identification key author's choice of character states impact algorithm performance? How does the difference in the minimum number of questions to identify various species influence algorithm performance?

We answer these questions via a computer simulation that exhaustively 'identified' all of the actual Jasper Ridge bird observations using our algorithm with a variety of parameter settings. The measure of performance for each setting of the algorithm is the number of questions required to identify a species being examined.

## Methods

We begin with a description of the experimental setup, after which we introduce the algorithm that computes the optimal decision tree from the historical data. We then present two alternatives for processing previously unobserved species. These algorithms are well known in the literature, but they have been applied primarily in sub-disciplines of Computer Science. Their application to species identification is new. In the results section we present the statistical analyses that answer the above research questions.

### Apparatus

**Figure 3:**
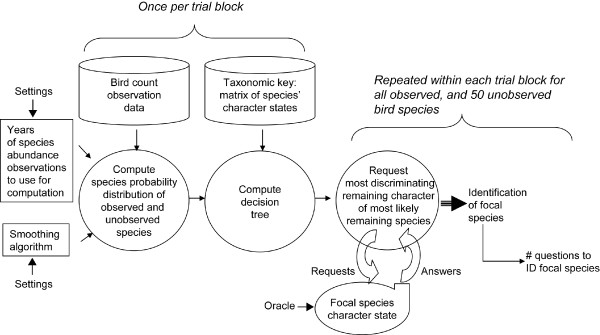
Conceptual flowchart of computer simulation setup to generate the number of questions required to identify a focal species. The process in the figure identifies one focal species at a time. Parameter settings (left) were varied in the following sets of experiments: (1) the number of years of historical observations and age of the years of historical observations were varied to measure their impact on the number of required questions. (2) To examine how best to account for previously unobserved birds we varied the probability smoothing algorithm used for the decision tree computation. For each focal species, the simulation requested character states from the oracle, which always provided the correct state. Once enough characters had been determined to uniquely identify the focal species, the number of required questions was noted and another species was selected as focal species. This process continued until all 104 birds that were observed at Jasper Ridge had been identified. For the smoothing algorithm experiment an additional random selection of 50 previously unobserved birds was also identified.

Figure 3 shows our experiment. The entire assembly operates on a desktop computer.

Each run of the simulation identified one focal species. One block of runs identified 104 birds that were previously observed in Jasper Ridge. Some blocks additionally identified 50 random birds that have not been seen at the preserve. One of two simulation parameters was varied across blocks of runs: (*i*) the amount and age of historical observations, and (*ii*) a choice of smoothing algorithm for modelling previously unobserved birds.

For each block of runs the chosen amount of historic bird count observation data was fed to the observation probability distribution computation (circle on left). This computation produced a probability for each of the observed birds, plus a single probability that was used for any previously unobserved species. Experimental manipulations controlled how much historic data was provided to the computation. We varied this information across 11 blocks of runs. Six blocks each provided one year's worth of observations (2000, 2001, 2002, 2003, 2004, 2005) to the algorithm. Other blocks of runs utilized two years of data per run (2004–2005), three years (2003–2005), four years of data (2002–2005), five years (2001–2005); and one block finally provided six years of observations to the computation (2000–2005). That is, each of 104 species observed at Jasper Ridge Preserve at least once between 2000 and 2005 were identified 11 times, each run being driven by a different set of historical observations.

In a separate computation we created a nearly perfectly balanced question tree. This process did not consider any historic data, and we call this configuration the *Static *condition. This condition was our base line against which we compared the *Biased *conditions that did use historical data when constructing the decision tree.

The right-most circle in Figure 3 processed all the runs within a block. This module used the probabilities and the character matrix database at the top of the figure to decide which sequence of characters to ask about. Acting as an oracle, the module at the bottom of the figure provided the proper state for any requested character. This module – one could think of it as a second computer – thus took the role of what in real deployment would be the human operator. An important difference between this module and a human being is that the oracle module never erred when providing the state of a character. Note that in *EcoPod*'s user interface we included a number of mechanisms that soften this assumption of correctness for human users. The system alerts the human operator of intra-species variance, and users can rate their confidence in the character states they provide [12].

Once the focal species of a run was identified, the required number of character questions was noted and the next focal species was processed. Note that this procedure does not simply measure the length of the shortest path from root to correct species. The experiment also measures how many questions the machine asks to *find *that shortest path.

Observation data consisted of six years of an ongoing repeated Bird Point Count study at JBRP. Birds were observed approximately 4 times a month by several expert birders following a strict observation protocol. Observations were conducted at 20 sites within JRBP, and data from all of these sites were pooled so that historical observations reflect species abundances for the whole preserve. We make the implicit assumption that the count of a species reflects its actual abundance, and we use the terms interchangeably. The bird species identification matrix was for > 700 western North American birds all of which were digitized by hand from the guide's paper format [18].

### Creating the Dichotomous Tree

As sketched in the Background section, two considerations enter the construction of the dichotomous tree from the matrix. The first is whether we assume uniform distribution of observations over all species, or whether we figure local history of actual observations into the construction of the tree. The second consideration is how to account for species that have not been observed in the geographic area before, but might be sighted in future field trips to the same region. We discuss both aspects here. Underlying the solutions to both issues is the probability of observing a given species in the future.

#### Computing Probabilities

Table 2 shows an elaboration of Table 1, adding hypothetical abundance observations. For each bird Table 2 shows its probability of being seen in the future, computed once under the assumption that each species is equally likely to be observed (a uniform distribution assumption), and again under the assumption that the probability of observing a species is impacted by the historical abundance of the species. This latter quantity is derived from maximum likelihood estimates (MLE).

**Table 2:** Hypo the tical set of observations and species characters. Two probability distribution assumptions are shown.

	Characters of taxonomic key				Probability mass	
			
Species name	Color	Slender	Bill length	Abundance (# of observations)	Uniform distribution	Maximum Likelihood Estimate (MLE)
Murres	White	No	Short	4	0.25	0.20
Gray Jay	Gray	Yes	Short	3	0.25	0.15
Egret	White	Yes	Long	11	0.25	0.55
Turkey	Gray	No	Short	2	0.25	0.10

Given the total of 20 observations of this example, the maximum likelihood estimate for Murres would be 4/20 = .2, that of Gray Jay would be 3/20 = .15, and so on. The next section describes how these probabilities are used to construct dichotomous trees used in the identification process.

#### Information Gain Maximization

The computation of dichotomous trees is a well-studied problem in Artificial Intelligence, where the construct is known as *decision trees *[19]. These constructs are a generalization of biology's dichotomous trees. The calculation of decision trees involves the notion of Information Gain. At each level of the tree, the algorithm chooses the character that maximizes the information gained (the most discriminative character). Maximizing information gain under a uniform distribution assumption in practice results in balancing the tree, minimizing its average depth.

Intuitively, the dichotomous tree is constructed top down from its root, which is the node at the top. That node has no parent nodes. The root node is created and is associated with one character. This character is chosen based on species probability. Under a uniform distribution assumption the character that eliminates close to 1/2 of the remaining rows is chosen. In Table 2 we see that 1/2 of the birds are white, while the other half are gray. Knowing the color therefore eliminates 1/2 of the rows. Asking about bill length would instead partition the space at a ratio of 1 to 3. Color would therefore be used as the root node.

Using MLE instead of uniform distribution, Egrets are about three times more likely than the other entries in the matrix. For this case bill length would move higher in the list of good characters to ask early. Information gain theory formalizes this use of probabilities for the construction of optimal dichotomous trees. Please see Additional file 1 for further information. In Additional file 1, we work through the mathematical details of character selection in terms of this running example.

Once the root node has been associated with the 'best' character, a child (next tier) node is created for each state that the character can take on. For each of these child nodes the process of associating a character is repeated as per the initial root node. The process is repeated until all terminal (lowest tier) nodes are species. Expressed as an algorithm this procedure runs as follows:

1. Begin at the root node.

2. For each node, determine the character with the highest information gain that is not used in an ancestor node.

3. Add a child node for each possible state of that character.

4. For each of these new nodes, attach a list of species that follow down the tree to that node.

5. Mark any of the species that are now uniquely identified as leaf nodes.

6. Go back to step two if not all terminal nodes are individual species.

Note that the use of information gain for constructing the question tree is elegant in that it only relies on the probabilities that are derived from observations. These probabilities in turn reflect very complex underlying influences, like local weather patterns, the prevalence of predators, and availability of food sources. It would be very difficult to model all these factors explicitly.

On the other hand, whether using a uniform distribution assumption or not, this algorithm takes into account only the species that have been observed in an area. Of course, there is also a chance that a previously unseen species invades or just was not observed before by chance. The next section introduces two alternatives from Computer Science practice that could be applied to address this shortcoming. We will later examine which of these two is the better choice.

#### Probability of Unseen Species

Using Maximum Likelihood Estimates would result in a probability of zero for previously unseen species (species not present in the historical observation data). The system would thus never enable successful identification of a species that is invading a new habitat, or was too rare to have been previously observed. The process of modelling the probability of unseen species is an example of 'smoothing,' because the probability mass needs to be 'smoothed,' or spread among seen and unseen events. Probability mass is the discrete-variable equivalent to the integral of the probability density function for continuous random variables. Literature addressing this issue is found, for example, in the area of Natural Language Processing [20-22], where computer programs attempt to predict the co-occurrence of words in speech or written text from previous observations. The reason for the existence of many smoothing algorithms is that their efficiency depends on the corpus that is being manipulated. The question is therefore whether the distribution of species observations is similar enough to the occurrence of words in natural language that the same smoothing algorithms can be applied in both areas. The two techniques are known as Laplace, and Good-Turing smoothing.

#### Laplace (Add-One) Smoothing

Laplace smoothing increases each observed frequency by 1. Therefore, species that have never been seen before are now assumed to have been observed exactly once. Species that have been observed *r *times in the past are now assumed to have been seen *r+1 *times. Therefore, probabilities are computed as follows:

$$P(Species\;X)=\frac{(Number\;of\;Observations\;of\;X)+1}{Number\;of\;Observations+Number\;of\;Species\;Found}$$

or concisely:

$$P(x_{r})=\frac{r+1}{\sum_{r}(r*N_{r})+\sum_{r}N_{r}}$$

where *r *is the historically observed abundance of species *x*, and *N*_*r *_is the number of species with abundance *r*.

The Laplace method has the advantage of being straightforward to describe and implement. However, in Natural Language Processing, Laplace smoothing has been found to overestimate the probability of unseen events.

This overestimation problem of Laplace smoothing has led to the development of alternatives. One family of algorithms from the literature is known as Good-Turing smoothing. In the following paragraphs we very briefly summarize the underlying concept in terms of species identification. For details please see Additional file 2 and [21], where the procedures are discussed in the context of natural language processing.

#### Good-Turing Smoothing

Like Laplace, Good-Turing smoothing modifies observed abundances *r*, discounting them by some amount. However, the method takes a more subtle approach. Intuitively, the technique notes how abundance observations change across species. A linear regression then fits a function through these variations of abundance. Using this function instead of the actual abundances to compute probabilities results in very abundant species contributing a bit less to the probability than they normally would. This discounted probability mass is effectively assigned to the never-seen species. This approach is a more controlled redistribution of probability than the crude add-1-everywhere Laplace approach. However, the algorithm is also more complex. The steps are as follows:

1. Rank the frequency of observations: count how many species were observed exactly once and note this quantity as *N*_1_. Count how many species were observed twice and note that quantity as *N*_2_, and so on.

2. Once ranking is complete, use local averaging to create non-zero values for any *N*_*r *_that are zero (frequencies at which no species was observed).

3. Fit linear regression to the resulting ranked list.

4. Compute the probability of seeing an unobserved species as $\frac{N_{1}}{N}$, with *N *being the total number of observations.

We describe the details of this process in Additional file 2.

#### Summary of Probability Considerations

Use of historical observations of species abundance to calibrate the identification process requires consideration of unseen species. We described assigning probability mass to previously unobserved species using two existing smoothing techniques that are popular in natural language processing. The question is whether the Good-Turing algorithm needs to be used for a dataset like the Jasper Ridge bird counts when computing probabilities for the dichotomous key, or whether the simpler Laplace method suffices. We applied both algorithms to our experimental setup and measured the resulting number of questions for both seen and unseen species.

## Results

We now present the data analyses of our experiment separately for each of the research questions we introduced earlier. For use in some of the analyses we computed the mean of all bird abundances over the six years of observations. We call the result *MeanAbundance*. We use the notation *yyyy_n *to indicate an observation year *yyyy *with *n *previous years' of data included. For example, 2005_3 means three years' of observations, the most recent being 2005: 2003, 2004, and 2005. All historical computations used Laplace smoothing.

**Figure 4:**
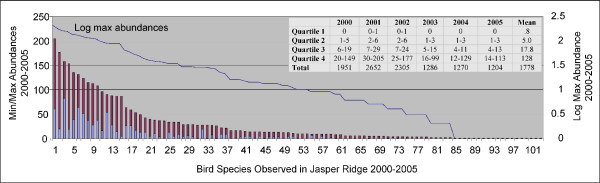
Min-Max overview of observation data. Each bar represents one species. The height of a stacked bar corresponds to the maximum one-year count during the 2000–2005 time span. The lower stack indicates the lowest one-year count during this period. The line (corresponding to the right-hand scale) shows the log of the mean abundance of each species over the six year period. The table inset shows the abundance quartiles during each year. The 'Mean' column contains the quartile cut-offs for the six year abundance mean.

Figure 4 provides an overview of the Jasper Ridge observation data. The abscissa shows numeric codes for all 104 observed species. The left ordinate shows species counts, while the right hand ordinate is a log scale. The top of each stacked bar marks the maximum yearly count of the corresponding species. For example, the left most bar corresponds to the Chestnut Backed Chickadee (this species name is abscissa ID 1 in the chart). During the species' most abundant year it was observed 205 times. The lower stack marks the count of the species' least abundant year, in this case 61. The line in Figure 4 shows the log of each species' mean abundance over the six years (right hand ordinate).

The table inset of Figure 4 shows the quartile ranges for abundances during each year. For example, during 2000 the rarest 25% of Jasper Ridge birds were not seen. During 2001 the rarest 25% were seen either not at all, or once. The lowest row shows the sum of all counts during the respective year. The 'Mean' column shows quartile cut-offs for the mean abundance across all six years. We call these cut-offs the *MeanQuartiles*.

The first research question we address is whether historical abundance observations provide any benefit at all for minimizing the average number of questions asked to identify a species. The inclusion of such information in the probability computations is contrasted with the base case of the uniform distribution assumption (*Static*).

### Does Observation History Add Significant Benefit?

**Figure 5:**
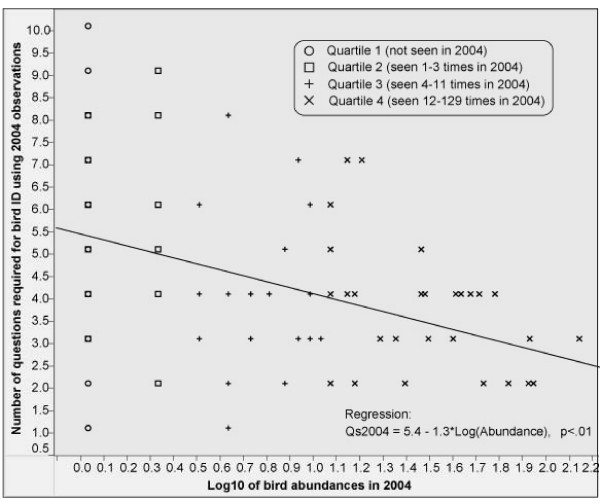
Number of questions the biased algorithm required when provided with observations from 2004. The abscissa shows log abundance. High abundance species require fewer questions.

The nature of the biased algorithm would have us expect a strong impact of bird observation abundance on performance. This built-in dependency is illustrated in Figure 5 where abundance is plotted against the number of questions the biased algorithm settings required when provided with observation data from 2004. We see that low abundance birds sometimes require very few, but also up to 10 questions to identify.

High abundance birds consistently require lower numbers of questions to ID. For formal verification we used the mean abundance over six years to partition the birds into quartiles, from rare (1^st ^quartile) to common (4^th ^quartile). A repeated measure ANOVA comparing the number of questions required when the algorithm was run with observations from 2000, 2005, and 2005_6 was conducted, with MeanQuartile as a between-subject factor. The result verified the expected interaction between runs and quartiles (F_7,234 _= 2.4; p < .02). In addition, the highly significant regression equation predicting the number of questions from log abundance is included as further verification at the bottom of Figure 5. Given this predictable sensitivity of the algorithm to abundance we conducted all subsequent tests separately by quartiles.

**Figure 6:**
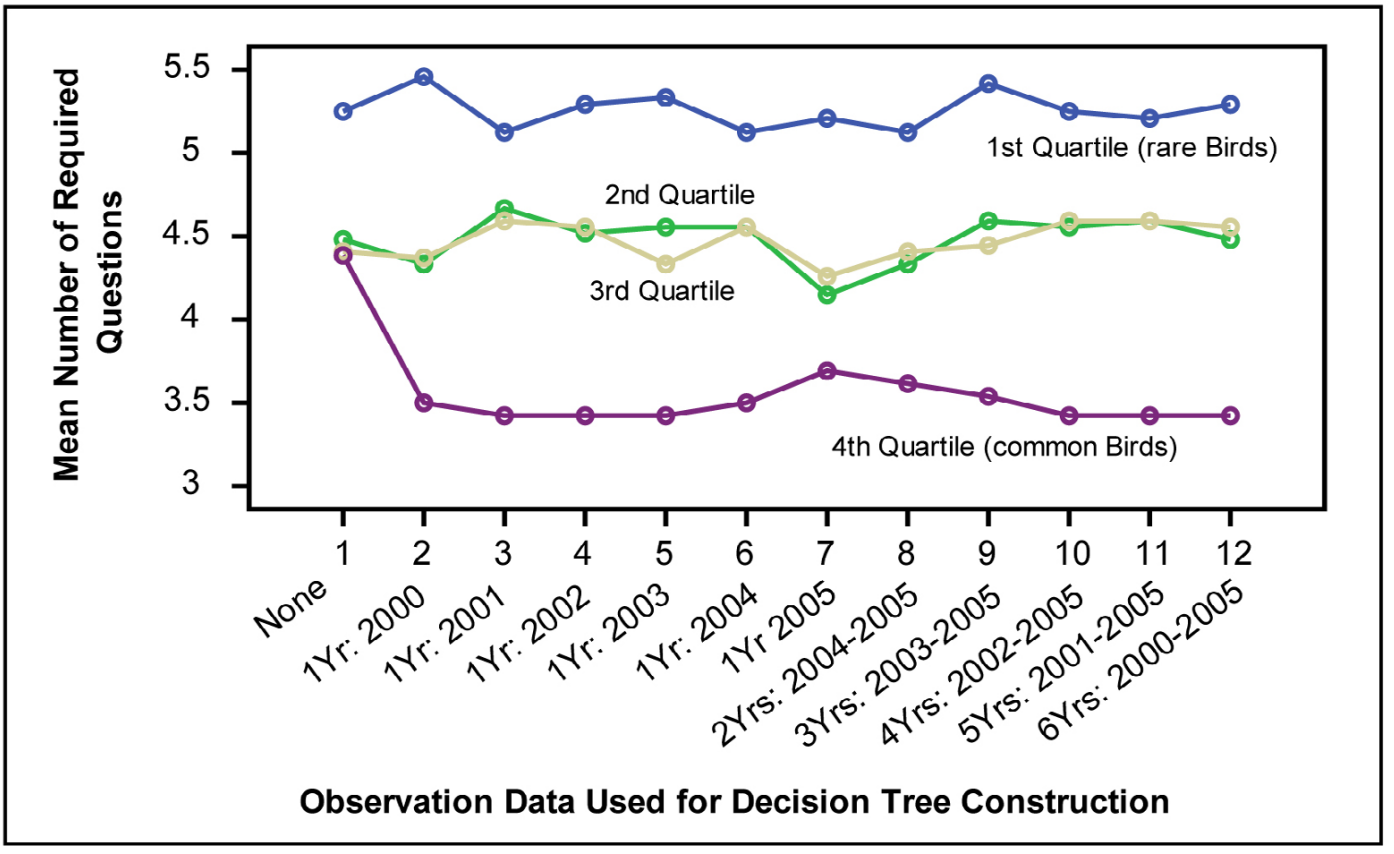
Performance of algorithm when provided with observations from different years and from different time spans. Each line shows results for a different abundance quartile.

Figure 6 shows an overview of the performance results partitioned by the amount of data that was made available to the decision tree construction algorithm.

Each line shows the mean number of questions required to identify one MeanQuartile of the birds when the algorithm was provided with observations from different years as indicated along the abscissa. The left most set of four (vertical) measurements corresponds to the *Static *condition when no observations are used for the decision tree construction. The five vertical sets of data points on the right show results when the algorithm was given access to several years' worth of observations (2005_1–6).

The answers to several of the research questions reduce to examining significance of differences between portions of Figure 6:

• *Does abundance information help? *↔ Is the difference between the four *Static *data points and the other data points significant?

• *Does it matter which year is used for observations? *↔ Are the differences between vertical sets 2 through 7 significant?

• *Does it matter how many years of observations are used? *↔ Are the differences between sets 7 and 12 significant?

• *Are rare birds unduly disadvantaged? *↔ For quartile 1 only, are any of the differences between *Static *and the remaining measurements significant?

We performed repeated measure ANOVAs separately for each MeanQuartile set of species. The independent factor was the amount of information provided to the algorithm; its levels correspond to the abscissa labels of Figure 6. The dependent variable was the mean number of questions required for identification. In each case we performed contrasts that compared each result for biased algorithm settings against *Static*.

For MeanQuartiles 1–3 no differences were significant. For MeanQuartile 4 the overall ANOVA measured F_3.5,88.7 _= 6.7; p < .01 (Greenhouse-Geisser corrected). Table 3 shows results of the contrasts, and means with standard deviations.

**Table 3:** Contrasts in 4^th ^MeanQuartile results of *static *against each of the biased algorithms.

***Static *vs...**	***Static***	**2000**	**2001**	**2002**	**2003**	**2004**	**2005**
**F**_**1,25**_	n/a	14.7; p < .01	17.2; p < .01	16.3; p < .01	15.4; p < .01	11.9; p < .01	7.5; p < .02
**Mean ± SD n = 26**	4.4 ± 1.7	3.5 ± 1.3	3.4 ± 1.3	3.4 ± 1.2	3.4 ± 1.3	3.5 ± 1.4	3.7 ± 1.2

***Static *vs...**	**2 Years**	**3 Years**	**4 Years**	**5 Years**	**6 Years**		

**F**_**1,25**_	10.0; p < .01	10.7; p < .01	14.7; p < .01	14.7; p < .01	14.7; p < .01		
**Mean ± SD n = 26**	3.6 ± 1.2	3.5 ± 1.2	3.4 ± 1.2	3.4 ± 1.2	3.4 ± 1.2		

The top portion of Table 3 contrasts the single-year runs against *Static*. The lower portion shows the comparisons between *Static *and the multi-year observations results (2004–2005, 2003–2005, etc.). Additionally, repeated contrasts were performed to compare all horizontally neighbouring results in Figure 6 (other than *Static*).

Very few differences were significant. These were:

• MeanQuartile 1-Two Years vs. Three Years: F_1,23 _= 4.3, p < .05.

• MeanQuartile 2-

◦ 2004 vs. 2005: F_1,26 _= 5.2; p < .03,

◦ Two Years vs. Three Years: F_1,26 _= 6.6; p < .02.

Note that the differences in the mean number of questions for these measurements are very small: The difference in mean for the significant MeanQuartile 1 contrast was .29 questions. The differences between the MeanQuartile 2 results were 0.4 and 0.3, respectively.

While ANOVA works well for the above analyses, its disadvantage in this situation is that a single quartile partitioning, namely MeanQuartile, must be used across all analyses. Recall that the MeanQuartile cut-offs are based on the counts averaged across six years. In truth, the quartile cut-offs are different each year. We concluded above that only the 4^th ^quartile, most abundant species afford an advantage over *Static *under the biased settings. In order to verify that this conclusion is valid for each of the years' quartile cut-offs, we repeated the ANOVA six times, each time using the cut-offs from a different year. In each case the 4^th ^quartile was clearly where the biased settings outperformed *Static*. In several cases the biased advantage extended into the 2^nd ^and 3^rd ^quartiles as well.

We conclude that the algorithm indeed reduces the number of questions required for identification, that this advantage only accrues for common species, and that neither the number of observation years used, nor the choice of years between 2000 and 2005 impacts this result for the Jasper Ridge Preserve bird counts of that time period.

### Penalty for Rare Species

**Figure 7:**
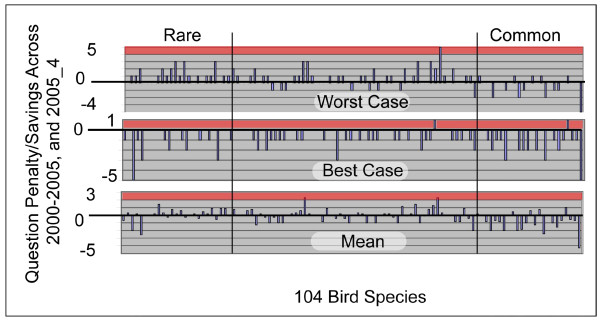
Detailed performance of biased runs against *Static *under three scenarios. For each of the 104 observedbird species the charts compare the number of questions required for *Static *with the number of questions required by one ofseven algorithm settings: 2000–2005, and 2005_4. Bars above the zeroline indicate that the biased runs required more questions than *Static*. Bars below zero indicate that *Biased *outperformed *Static*. The ordinate scale is number of questions required beyond *Static *(positive bars) or saved against *Static *(negative bars). The top panel compares *Static *against the worst performing biased setting for each species. The middle panel compares *Static *against the best performing setting, and the bottom panel compares against the mean of all seven biased settings for each species. Note that the panels have different ordinate scale ranges (5→-4, 1→-5, and 3→-5).

The lack of significant differences between *Static *and the biased runs for 1^st ^quartile species answers the question about whether the biased methods unduly handicap identification of rare species: the algorithm does no worse for rare species than the standard approach. Figure 7 adds a visual for details under three scenarios.

The figure shows how many more, or fewer questions the seven biased settings 2000–2005, 2005_4 require compared to *Static*. Since only one bar can feasibly be displayed for each species (rather than seven), we show three scenarios. The worst case scenario selects for each species the worst performing of the seven biased settings for the comparison against *Static*. The best case scenario (centre panel) instead chooses the best performer for each species, and the bottom panel shows results when *Static *is each time compared to the mean of the questions required by the seven biased settings.

Each bar provides the comparison information for one of the 104 Jasper Ridge bird species. A positive bar indicates that the biased settings required the indicated number of questions beyond *Static *for the respective species. A negative bar indicates that the biased settings performed better by the indicated number of questions. That is, negative bars are 'good.'

For example, in the best case only very few species would require more questions than *Static *as there are few positive bars. Note that in the bottom panel many bars are negative for common species, consistent with ANOVA results of previous sections.

### Intrinsic Ease of Identification

We examined two aspects of the data to gain partial insight into how intrinsic ease of identification impacts the number of questions that need to be asked towards identification of a species. The first data aspect we examined is the matrix density, the per species number of non-empty entries in the bird identification matrix. The second is the minimal number of questions the algorithm could possibly ask to identify each bird.

#### Sensitivity to Matrix Density

Species identification matrices will differ across both species and matrix authors. One such difference is the number of characters that are specified for each taxon (row). In our North American birds matrix the average number of non-empty cells per row was 12, with a range of 3 to 31 cells. In order to obtain a rising measure of *Simplicity *for each bird we divided this count into 100. For example, a bird for which our matrix specified states for 12 characters received a *Simplicity *score of 100/12 = 8.3.

To test whether the *Simplicity *measure impacts the biased algorithm runs we computed a repeated measure ANOVA for the eleven biased settings 2000–2005, 2005_2–6, with *Simplicity *as covariant. No interaction was detected between the covariant and the number of questions required for the bias settings. We conclude that at least for this North-American bird matrix the algorithms are not sensitive to the row densities.

#### Sensitivity to Ease of Identification

In order to extract the minimal, best case number of questions that the biased algorithm settings could elicit for any given bird we computed a *MinNumQs *value for each species. This value was obtained by in turn artificially setting the probability of each bird high, even if its abundance was low. We then noted the number of questions that the algorithm generated in turn for each such artificially high-biased species.

For *MeanQuartiles *1 and 2 strong correlations were in evidence between *MinNumQs *and each of the eleven biased settings. The Pearson coefficient for all these correlations was close to .7, p < .01. For *MeanQuartiles *3 and 4 no significant correlations were found. However, the same correlation pattern was found to hold between *MinNumQs *and *Static*. The relative performance of *Static *and the biased settings was thus unaffected by *MinNumQs*.

We verified this conclusion by normalizing all our measured results to neutralize the impact of *MinNumQs*. For each species we divided the number of questions generated under *Static *and each of the biased settings by that species' *MinNumQs*. We then repeated all the previously described analyses for these normalized results. All the conclusions remained unaltered.

We conclude that the intrinsic differences of how difficult various species are to identify does not in practice influence how much better or worse the biased methods perform relative to *Static*.

### Does the Smoothing Algorithm Impact Performance?

Our implementations of both Laplace and Simple Good-Turing (SGT) smoothing allowed us to compare performance under both algorithms. We computed abundance probabilities for each of the matrix species, both seen and previously unobserved at Jasper Ridge. For all of the 104 previously observed bird species, and 50 randomly selected never locally spotted birds we computed the number of questions required for identification.

**Figure 8:**
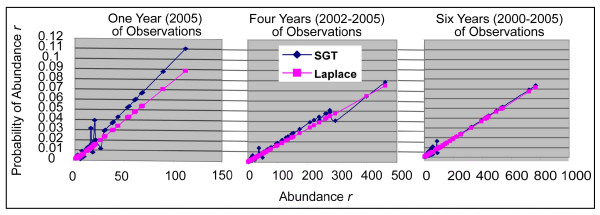
Probabilities generated by Laplace and SGT smoothing for1, 4, and 6 years of observations.

Figure 8 provides an intuition for the algorithms' generation of probabilities. As the number of total observations increases (from the left panel towards the right), SGT probabilities grow more and more similar to Laplace estimates. The total probability apportioned to seen species corresponds to the area under the graph. SGT's smaller than Laplace's set-aside for unseen species is reflected in SGT's steeper slope for one-year observations. The SGT algorithm in fact loses validity beyond four years of observations. At that point the slope of the *Z*_*r *_regression exceeds -1 (see [22] for an explanation of this slope having to be less than -1). At four years the slope is just about at that critical threshold. This apparent instability of species observation data sets in the context of SGT amplifies the need to understand whether the more sophisticated SGT is actually required for minimizing questions.

We performed a repeated measures ANOVA over the number of questions required for both Laplace and SGT, for both one year's and five year's worth of observations. Table 4 shows results for each abundance quartile separately. Each cell is subdivided into four quadrants, each one holding a result for one quartile. Italicized values are significant at *p < .01*. Other values are significant at *p < .05*. The left portion of Table 4 shows the per-quartile mean of the number of required questions for each of the smoothing algorithms and amount of observation data. The F values are results of planned contrasts. Non-significant comparisons are marked with 'ns.'

**Table 4:** F-value results of comparing Laplace and Simple Good-Turing smothing for single-year (2005_1) and five-year (2005_5) observation amounts. Cells are subdivided to hold results from each abundance quartile separately; quartile order is clockwise (see top left of table). The right-hand portion of the table summarizes means for all conditions. In 2005, 31 of the 104 Jasper Ridge species were not observed. During the five-year span only one species remained unobserved.

**Legend for Cell Partitions:**		**Laplace 5 Years F values**		**SGT 1 Year F values**		**SGT 5 Years F values**		Mean Number of Questions ± SD	
								
*Q*4:F_1,25_	*Q*1:F_1,26_								
								
*Q*3:F_1,26_	*Q*2:F_1,25_								
**Laplace 1 Year**		ns	ns	4.5	*9.3*	ns	ns	3.7 ± 1.1	5.2 ± 2.3
		ns	ns	ns	ns	ns	ns	4.3 ± 1.5	4.0 ± 1.9
**Laplace 5 Years**				ns	*8.3*	ns	ns	3.5 ± 1.2	5.4 ± 2.6
				ns	ns	ns	ns	4.6 ± 2.0	4.4 ± 2.3
**SGT 1 Year**		Italicized F values: *p *< .0.1, others: *p *< .05.				ns	*11.4*	3.5 ± 1.1	6.5 ± 3.3
						ns	ns	4.3 ± 1.4	4.4 ± 2.5
**SGT 5 Years**								3.5 ± 1.2	5.1 ± 2.3
								4.4 ± 1.9	4.4 ± 2.4

Note that during 2005, 31 of the 104 species that are sometimes seen at Jasper Ridge were not observed. Throughout the 2001–2005 period against which we compare, only one species was not seen. That is, the 2005_1 settings contain more rare birds than the 2005_5 settings.

From Table 4 it is evident that whenever Simple Good-Turing smoothing is applied to the identification of unseen species the results are worse than when Laplace smoothing is used. For example, comparing the one-year Laplace with one-year SGT, first quartile birds required 5.2 questions using Laplace, but 6.5 questions using SGT (F_1,26 _= 9.3, p < .01). When abundant species are being identified, the difference between Laplace and SGT tends to disappear. The Laplace one-year/SGT-six-year differences are not significant for any quartile.

We explored the impact on rare species further by having the algorithm identify 50 birds that were never seen in Jasper Ridge, using the probabilities from the above experiment as biased settings: Laplace 2005_1, Laplace 2005_5, SGT 2005_1, and SGT 2005_5. That is, the probabilities that guided the decision tree construction were computed from the abundances of observed birds, but the 50 identifications always had to rely on the probability that the smoothing algorithms set aside for never seen species. The resulting number of required questions (mean ± SD) were, respectively, 5.8 ± 2.6, 7.3 ± 3.0, 6.6 ± 3.2, and 7.4 ± 3.0. The 50 species identified for all four runs were each time randomly selected from among the roughly 250 species that were available in our key but had not been seen at Jasper Ridge. A oneway ANOVA was significant (F_3,199 _= 3.0, p < .05). Posthoc tests showed the only significant difference to be the additional 1.6 questions required by six-year SGT when compared to the one-year Laplace.

We conclude that for rare species the Laplace approach is more reliable than Simple Good-Turing.

## Discussion

Above we reported results from three directions of exploration. First, we investigated whether the number of observation years and the age of observations impacted the performance of the identification algorithm. Second, we examined how some characteristics of our identification key influenced the number of required questions. And third, we compared two smoothing algorithms that impact how well the identifications work for previously unobserved species. In the following sections we add some thoughts to these results.

For all our results we stress that they are based on one particular data set from the relatively protected confines of a biological preserve. Further studies will be required to evaluate the impact of high instability in the ecosystem on the algorithm's performance.

### Number and Choice of Observation Years

Results showed clearly that the inclusion of historical observations can significantly accelerate an identification tool. However, this benefit only accrues once a sufficient amount of observation data is available. That amount corresponds to the top 25% most abundant species. In our data set this means that birds seen at least 12 times during a one-year period (4^th ^quartile cut-off for 2004) benefit significantly from the biased approach. Those species are, of course, precisely the ones that are the most important to identify quickly, because they make up the bulk of census activities.

Surprisingly, the number of years of accumulated data was not found to significantly impact the algorithm's performance. A single year's observations performed at the same level as six year's worth of data. Also, no difference was found for which year's observations were used to run the algorithm. This robustness is good news because observations are expensive to gather and maintain. Within limits, fluctuations in species populations do not greatly impact results.

Looking at the modest magnitude of differences between *Static *and the biased results one might wonder whether an advantage of one question saved per identification is worth the trouble. Notice, however, that this savings is multiplied across many sightings and all observers that participate in a census.

### Impact of the Key's Characteristics

Fortunately, the broad mixture of bird matrix row densities in our digitized key did not impact the algorithms. This result suggests that matrices by different authors and for diverse species focus will be amenable to our question generation algorithms. This assumption remains to be tested with other matrices and species, as well as with other observation datasets.

We were surprised that the minimum number of questions (*MinNumQs*) did not interfere with our results. We had expected that we would need to normalize results to control for this species-specific quantity. It turned out, however, that the correlations between *MinNumQs *and the algorithm outputs were very uniform across all experimental conditions, and most importantly affected the *Static *condition equally. This result again is good news because it limits the amount of work required when introducing a new key into the identification system. No analysis of minimum question requirements is needed.

### Probability Smoothing Techniques

Results of the smoothing technique analysis clearly imply that for this dataset at least Laplace smoothing is the algorithm of choice precisely because of its reputation for overestimating unseen observations. Our analysis confirms this tendency for excessive apportioning of probability mass to unseens, in that Laplace clearly improves the performance for never sighted species. Given that Laplace does not in turn hurt performance for observed species, the complications of Simple Good-Turing seem unnecessary and Laplace is the smoothing method to use.

### Limitations and Future Work

Two sets of limitations and consequent need for additional work apply to the material discussed here. One set concerns the data set and experiment, the other concerns more broadly our EcoPod tool.

Our study was based on bird observations in a preserve. One might argue that populations could change more rapidly in less protected environments, or for organisms other than birds. It would therefore be useful to repeat our experiment with historic data from other ecosystems. More than one year's worth of observations might be required for those cases, even though we found that a single year sufficed.

Similarly, it would be useful to repeat our experiments for a different identification key, preferably for species other than birds. Even though we did test for dependencies of the algorithm's efficiency on the key matrix's distribution of sparsity and on the ease of identification for each species, these tests were by necessity limited to the matrix we used. Repeat experiments with other keys would solidify our findings.

Regarding EcoPod and its efficacy, neither of our ease of identification measures captures the *practical *difficulties of observing particular characters in the field. The measures do not, for example, take into account the difficulty of measuring the length of a squirrel's hair at 100 ft, as compared to evaluating the animal's colour. Factors like those would need to be captured explicitly, or through the device observing which characters users choose to specify during a number of field excursions. EcoPod users are not forced to answer questions in order. They may choose to specify characters further down the question list early. In an improved tool their choice would then calibrate the characters that are preferentially solicited of the user in the future.

Similarly, environmental cues could be worked into the device's question sequencing. For example, geographic location and season might be used in the biasing of probabilities that guide the decision tree construction.

A field worker's skill and experience could further be used to influence EcoPod's behaviour. For example, the device could ask some questions that are not strictly required for identification, but would help avoid misidentification when two species are similar. Such a question could, for instance, inquire about a character that should *not *be of a particular state, given the user's answers so far.

We next discuss related work and then present concluding remarks.

## Related Work

The cross-disciplinary nature of this work induces several strands of prior work. We cover bio-diversity and Computer Science related work separately.

### Biodiversity

Observations by volunteers have resulted in datasets which have been invaluable to scientific research and conservation efforts. About 480 scientific papers have been written on the 105 years of Christmas Bird Count observations (CBC) [23], informing topics such as the range of bird species, spread of invasive species, change in population sizes and species extinctions.

The Fourth of July Butterfly Count (FJC) [24] has provided crucial data on abundance and population dynamics of common species e.g. [25-27], as well as on monarch butterfly range and migration, and changes in population size with continued destruction of over-wintering habitat [28]. Such data have informed conservation needs, such as the recent identification of areas with stewardship responsibility for maintaining high levels of species abundance e.g. [29]. The case for citizen participation is also made in [30].

In addition to mass-participation census taking, continuous species observations can now be collected into online databases. One example, the Calflora project, has collected over 850,000 observations of more than 7,600 plant species in California. Websites supporting the CBC and FJC have also created databases for users to contribute observations, including *eBirds *[31] and *Butterflies I've Seen *[32] respectively. Observations collected by a single conscientious individual have been invaluable for determining the impacts of global warming on birds [33].

### Computer Science Related Work

Decision tree theory is explained in [19], where basics of information gain theory are also covered. Techniques for probability smoothing are discussed in [21,22].

A number of computer based species identification tools exist. Many tools are direct carry-overs of paper-based field guides to electronic versions (e.g. eBird [31] and *Handheld Birds *[34]. They are essentially scanned pages of a field guide that harness the power of the PDA by incorporating bird calls that cannot be available with the paper version. Species identification is achieved in those systems through pictorial recognition, like in paper field guides, rather than via a question and answer-based taxonomic key. *Handheld Birds *provides a minimal form of taxonomic key by allowing the user to sort species by providing the states of four characters. *CyberTracker *and *Handheld Birds *are the only PDA-based identification tools that allow observations to be contributed to a central database for use in scientific research. *CyberTracker *[35] is unique in providing an icon-driven interface that handles only pictorial information. This approach allows non-literate contributors who are skilled in the identification of animals to record observations of mammals. These observations are reported to a central database for use in scientific research. *PalmKey *is a PalmOS based identification tool that is freely available and allows users to create their own key databases [36].

Within Computer Science research our work is in the tradition of optimizing user interfaces based on artificial intelligence techniques and usage patterns. There has been considerable research in the Computer Science community on systems that make use of accumulated data from different users, such as visitors to a Web site, and that 'track' user actions to optimize future renditions of the user interface. In our system, historical abundance measures are analogous to such data from many different users in the field.

Research into multi-user data systems has grown after the advent of the World-Wide-Web, since it has now become possible to collate and mine usage patterns from users spanning diverse geographical regions. Several multi-user tracking systems exist including: *Context-Aware Proxy based *System (CAPS) [37], *SurfLens*, [38], and *ProfBuilder *[39]. These systems track user browsing habits, navigation histories or site usage information respectively to make recommendations to the user or as a basis for collaborative filtering.

## Conclusion

We showed the algorithms that underlie our EcoPod, an in-field species identification tool. In particular, we focused on showing how the tool minimizes the number of questions it asks of the user during the course of an identification.

The main source of our optimization is the frequency of past observations in the field where the device is deployed. The more often a species was observed in the past, the more the tool favours solicitation of characters for that species over characters that are discriminants for less frequently sighted species. We employed Computer Science algorithms, particularly from the theory of decision tree learning, and smoothing techniques that are popular in the area of natural language processing.

We tested the question generating algorithm on point count bird observations that were conducted at Stanford's Jasper Ridge Biological Preserve. The data covered the years 2000–2005. The algorithm generated questions driven by an identification key of North American birds, which we transcribed from its original book form to an online matrix.

We showed that the question generating algorithm is not sensitive to how many of the six years' worth of observation data are supplied as input. Even one year's worth of data made all the difference. We also showed that, at least for our bird observations at the Jasper Ridge Preserve, the age of the data (within those six years) did not impact the algorithm's efficiency. Observations from 2000 were just as valuable as observations from 2005.

The key matrix's varying density of characters for each bird species had no impact on the algorithm. Neither did differences in the minimum number of required questions among the species affect the algorithm differently than it affected the baseline alternative. That is, some species do require more questions, no matter how the question sequence is constructed.

Finally, we showed that the use of Laplace smoothing during the calculation of probabilities for the future observation of each species works well. This result contrasts with findings in other areas, like Natural Language Processing, where variants of the Good-Turing algorithm are preferred.

The inclusion of the public in the collection of observation data is crucial if convincingly large and geographically diverse data sets are to be accumulated quickly. A challenge in the way of such public participation is quality assurance. EcoPod is one tool that attempts to enable convenient and reliable collection of observations.

## Authors' contributions

YYY wrote the EcoPod implementation. AM and AP worked on the simulations and algorithms. JS worked on design features and all the biological aspects. Throughout the project, though, the four authors operated as a team, contributing to all aspects of the work. All authors read and approved the final manuscript.

## Supplementary Material

Additional file 1 Appendix 1: information gain. This appendix describes the Information Gain algorithm in detail with examples. Click here for file

Additional file 2 Appendix 2: Good-Turing smoothing. This appendix describes the Good-Turing smoothing algorithm in detail with examples. Click here for file

## References

[B1] Olson DM, Dinerstein E, Powell GVN, Wikramanayake ED (2002). Conservation Biology for the Biodiversity Crisis. Conservation Biology.

[B2] Luck GW, Ricketts TH, Daily GC, Imhogg M (2004). Alleviating spatial conflict between people and biodiversity. Proceedings of the National Academy of Science.

[B3] Hughes JB, Daily GC, Ehrlich PR (1997). Population Diversity: Its Extent and Extinction. Science.

[B4] Swengel AB (1995). Population fluctuations of the Monarch (Danaus plexippus) in the 4th of July Butterfly Count 1977-1994. American Midland Naturalist.

[B5] McLaughlin JF, Hellmann JJ, Boggs CL, Ehrlich PR (2002). Climate change hastens population extinctions. Proceedings of the National Academy of Sciences of the United States of America.

[B6] LeBaron GS (2005). The 105th Christmas Bird Count. American Birds, The 105th Christmas Bird Count.

[B7] Battersby JE, Greenwood JJD (2004). Monitoring terrestrial mammals in the UK: past present and future, using lessons from the bird world. Mammal Review.

[B8] Battersby J (2005). Engaging with volunteers and managing volunteer networks.

[B9] Newman C, Buesching CD, Macdonald DW (2003). Validating mammal monitoring methods and assessing the performance of volunteers in wildlife conservation - "Sed quis custodet ipsos custoides". Biological Conservation.

[B10] Gaston KJ (2000). Global patterns in biodiversity. Nature.

[B11] Dallwitz MJ (1974). A flexible computer program for generating identification keys. Systematic Zoology.

[B12] Yu Y, Stamberger JA, Manoharan A, Paepcke A (2006). EcoPod: A Mobile Tool for Community Based Biodiversity Collection Building.

[B13] Morse LE (1971). Specimen identification and key construction with time-sharing computers. Taxon.

[B14] Dallwitz MJ, Paine TA, Zurcher EJ (1993). User's guide to the DELTA System: a general system for processing taxonomic descriptions..

[B15] Dallwitz MJ, Paine TA, Zurcher EJ (1995). User’s guide to Intkey: a program for interactive identification and information retrieval.

[B16] Lucid. http://lucidcentral.com/.

[B17] Osborne DV (1963). Some Aspects of the Theory of Dichotomous Keys. New Phytologist.

[B18] Farrand JJ (1988). Audubon Handbook: How to Identify Birds.

[B19] Chen SF, Goodman J (1996). An Empirical Study of Smoothing Techniques for Language Modeling.

[B20] Katz SM (1987). Estimation of Probabilities from Sparse Data for the Language Model Component of a Speech Recognizer. IEEE Transactions on Acoustics, Speech, and Signal Processing.

[B21] Gale WA, Sampson G (1995). Good-Turing Frequency Estimation Without Tears. Journal of Quantitative Linguistic.

[B22] Christmas Bird Count. http://www.audubon.org/bird/cbc/index.html.

[B23] Fourth of July Butterfly Counts. http://www.naba.org/counts.html.

[B24] Swengel AB (1990). Population Fluctuations of the Monarch (Danaus plexippus) in the 4th of July Butterfly Count 1977-1994. American Midland Naturalist.

[B25] Nagel HG, Nightengale T, Dankert N (1991). Regal fritillary butterfly population estimation and natural history on Rowe Sanctuary, Nebraska.. Prairie Naturalist.

[B26] Nagel H (1992). The link between Platte River flows and the regal fritillary butterfly. The Braided River.

[B27] Swengel AB, LaRoe ET, Farris GS, Puckett CE, Doran PD, Mac MJ (1995). Fourth of July Butterfly Count. Our Living Resources: a report to the nation on the distribution, abundance, and health of US plants, animals and ecosystems.

[B28] Wells J (2005). Boreal Forests in Canada and Alaska. American Birds, Summary of the 105th Christmas Bird Count.

[B29] Stevenson RD, Haber WA, Morris RA (2003). Electronic field guides and user communities in the eco-informatics revolution. Conserv Ecol.

[B30] eBird. http://www.ebird.org/content/.

[B31] Butterflies I've Seen, North American Butterfly Association. http://www.nababis.org/servlets/Sightings.

[B32] Ledneva A, Miller-Rushing AJ, Primack RB, Imbres C (2004). Climate Change as Reflected in a Naturalist's Diary, Middleborough, Massachusetts. Wilson Bulletin.

[B33] Russell SJ, Norvig P (2002). Artificial Intelligence: A Modern Approach.

[B34] Geographic N Handheld Birds; Mobile Interactive Field Guide. http://www.handheldbirds.com/.

[B35] CyberTracker. http://www.cybertracker.co.za/.

[B36] Webb C PalmKey version 1.0. http://www.phylodiversity.net/palmkey/.

[B37] Sharon T, Lieberman H, Selker T (2003). A zero-input interface for leveraging group experience in web browsing: January 12-15 2003; Miami, Florida, USA..

[B38] Fu X, Budzik J, Hammond KJ (2000). Mining navigation history for recommendation.

[B39] Wasfi AMA (1998). Collecting user access patterns for building user profiles and collaborative filtering.

