# Supplementary material for: Optimizations for the EcoPod field identification tool
Source: BMC Bioinformatics. 2008 Mar 17;9:150. doi: 10.1186/1471-2105-9-150 (PMC2322985; doi:10.1186/1471-2105-9-150)
Supplement: Additional file 1 — Appendix 1: information gain. This appendix describes the Information Gain algorithm in detail with examples. [file 1471-2105-9-150-S1.doc]

# Appendix 1: Information Gain

We provide here the underlying formulae of information gain and work through a calculation based on our bird example of Table 2. The first formula specifies the *entropy* of a given character. Entropy is a measure of variability in a random variable, or more specifically for our case, in the states of a character across species. The higher a character's entropy, the more evenly the states of the character are distributed across all species. The formula for entropy H of a character C is:

$$H(C)= -\sum_{j=all of C's states} p_{j}* \log_{2}(p_{j})$$

where *p_j_* is the probability that character C will take on state *j*. For example, in the bird matrix of Table 2 the entropy of the Color character is computed as follows:

Probabilities: *p_white_ =* 0.5; *p_gray_* = 0.5

$$H(Color) = -(0.5*\log_{2}(0.5) + 0.5*\log_{2}(0.5)) = 1$$

Similarly, the entropy of the species name is:

*p_Murres_* = 0.25; *p_GrayJay_* = 0.25; *p_Egret_* = 0.25; *p_Turkey_* = 0.25

$$H(Species) = -4*(0.25*\log_{2}(0.25) = 2$$

Identifying a species is equivalent to driving the species entropy to zero through limiting the available choice of species by requesting character states. The notion of *conditional entropy* captures this process mathematically. The related formula for a character *C* is:

$$H(Species|C)=\sum_{j=all of C's states} p_{j}*H(Species|C=j)$$

where *p_j_* is the probability that C takes on state *j*. The conditional entropy measures how much entropy is left if one were to know the state of character C. For example, to determine how much entropy the Species column retains if a bird’s color were known,

we examine the following table:

| **Color (j)** | **Prob(Color=j)** | **H(Species|Color=j)** |
| --- | --- | --- |
| White | 0.5 | -2*0.5*log_2_(0.5) = 1 |
| Gray | 0.5 | -2*0.5*log_2_(0.5) = 1 |

$$H(Species | Color) = 0.5*1 + 0.5*1 = 1$$

In contrast, the computation for bill length:

| **Bill (j)** | **Prob(Bill=j)** | **H(Species|Bill=j)** |
| --- | --- | --- |
| Long | 0.25 | -1*log_2_(1) = 0 |
| Short | 0.75 | -3*0.75*log_2_(0.75) = 1.6 |

$$H(Species | Bill) = 0.25*0 + 0.75*1.6 = 1.2$$

The final required concept is that of information gain. It is defined in terms of entropy reduction:

$$IG(Species | C) = H(Species) - H(Species | C)$$

For our example:

$$IG(Species | Color) = 2-1 = 1$$

$$IG(Species | Bill) =2-1.2 = 0.8$$

That is, asking for color (given equal probabilities over the species) gains more information than asking for bill length.

In contrast, consider the matrix in Table 5, were the distributions are not equal for all birds. This time, even though the Color character would again partition the resulting tree into two groups of two species, the information gain computation would direct the algorithm to inquire first about bill length. In this case,

$IG(Species | Color) = 0.47$, while

$$IG(Species | Bill) = 0.99$$

The higher information gain for Bill Length would trump the request for the Color character state. Intuitively, this decision is correct because the ‘neat’ symmetric tree that would result from placing Color at the tree’s root would ***not*** be balanced with respect to species abundance: A total of *0.45+ 0.45* = *.9* of the occurrence probability would be situated on the *White* portion of the tree. Only *.1* of the probability would be associated with the *Color=Gray* half of the tree.

The question sequencing algorithm runs through these information gain computations for the full matrix, and for all characters. Each time a character is selected, the computations are repeated for the remaining species.

# Index of Tables

| Species | Color | Bill Length | Distribution |
| --- | --- | --- | --- |
| Murres | White | Short | 0.45 |
| Gray Jay | Gray | Short | 0.05 |
| Egret | White | Long | 0.45 |
| Turkey | Gray | Short | 0.05 |

Table 1: Same bird population, but non-equal distribution
