# Supplementary material for: Optimizations for the EcoPod field identification tool
Source: BMC Bioinformatics. 2008 Mar 17;9:150. doi: 10.1186/1471-2105-9-150 (PMC2322985; doi:10.1186/1471-2105-9-150)
Supplement: Additional file 2 — Appendix 2: Good-Turing smoothing. This appendix describes the Good-Turing smoothing algorithm in detail with examples. [file 1471-2105-9-150-S2.doc]

# Appendix 2: Good-Turing Smoothing

Instead of adding 1 to each observed abundance *r* as Laplace smoothing does, Good-Turing smoothing computes for each *r* a replacement *r**, defined as,

$$r*=(r+1)\frac{N_{r+1}}{N_{r}}$$

where *N_r_* is the frequency of frequencies: the number of species that occur exactly *r* times. In set notation *N_r_* this is the cardinality of the set of all species that occur *r* times in the data:

$$N_{r}= |\{Species X|Count(Species X)=r\}|$$

The probability for a species that was observed *r* times is then computed as

$$\frac{r*}{N}$$

where *N* is the total number of observations.

In practice, this approach runs into a difficulty that is illustrated in Table 6.

Not all observation frequencies are represented, causing *N_r_* to be zero for some *r*. An averaging step eliminates this problem. The non-zero *N_r_* that bracket runs of *N_r_*=0 are averaged to account for the ‘missing’ *r*. This process leads to a new *Z_r_*, which can be fitted with a straight line equation, allowing computation of *Z_r_* from any *r*. Figure 9 shows a comparison between *N_r_* and *Z_r_* for our 2006 bird observations. The averaging process follows [5].

The new quantity *Z_r_* can now be used analogously to the original *N_r_* to compute *r***:

$r**=(r+1)\frac{Z_{r+1}}{Z_{r}}$, with species probability estimated as $\frac{r**}{N}$ after a normalization step to ensure that probabilities add to 1. For small *r* the basic *r** computation works well. We therefore use what [5] calls the Simple Good-Turing method, which applies *r** until the probabilities it generates are significantly different from the linear regression based *r***’s probabilities. At that point the *r*** are used for larger *r*.

In either case, the total probability mass set aside for unseen birds is estimated as $\frac{N_{1}}{N}$, that is the number of species seen exactly once as computed via the regression, divided by the number of observations.

One final detail around the described procedure emphasizes the need for examining the algorithm’s suitability in the context of biological species observation: A proof in [5] shows that the slope of the regression line above must be less than -1 for the algorithm to work properly. Without this constraint met, the algorithm would *add* probability mass to the observed species, rather than withholding it for the unseen species. The slope, of course, depends on the particular data set. For natural language processing applications this constraint is usually met. Validation for our application required the analysis we presented in the Error: Reference source not found section.

# Index of Figure Legend

Figure 9: The effect of averaging frequency-of-frequencies to eliminate zero values and enable regression.

# Index of Tables

| ***r*** | ***N_r_*** | **Species Examples** |
| --- | --- | --- |
| 1 | **40** | American Black Duck, Savannah Sparrow, Merlin, … |
| 2 | **10** | Rock Dove, Winter Wren, … |
| 3 | **5** | Downy Woodpecker, Mallard, Double Crested Cormorant, … |
| … | **…** | … |
| 11 | **1** | Pacific-Slope Flycatcher |
| 12 | **0** | **←: *N_r_=*0, so *r** cannot be computed** |
| 13 | **0** | **←** |
| 14 | **2** | Cliff Swallow, Purple Finch |

Table 1: Zero values for *N_r_* cause problems with the basic Good-Turing formula
